# Interaction energy flow paths analysis of PMSG-based wind power integrated systems during LVRT and its parameter adjustment strategy

**DOI:** 10.1038/s41598-024-62507-z

**Published:** 2024-06-15

**Authors:** Chao Xing, Xinze Xi, Xin He, Can Deng, Mingqiang Zhang

**Affiliations:** Electric Power Research Institute of Yunnan Power Grid Co., Ltd, Yunnan, 65021 China

**Keywords:** PMSG, Low-voltage ride through, Interaction energy path, Parameter adjustment strategy, Broadband oscillation, Wind energy, Electrical and electronic engineering

## Abstract

To solve the problem of oscillation instability in permanent magnetic synchronous generator (PMSG)-based wind power connected systems during low-voltage ride through (LVRT) process, a parameter adjustment strategy based on interaction energy path optimization is proposed in this paper. Firstly, a modular state-space model of PMSG under fault transient conditions is constructed, and the system is divided into five subsystems. Then, the dynamic energy function of subsystems reflecting the oscillation stability of the system is derived. Based on that, the dynamic energy flow path is described considering the introduction of LVRT control. On this basis, the interaction energy between LVRT control links and subsystems is analyzed, and the coupling mechanism of voltage support and damping characteristics in the LVRT process is explained. Further, aiming at the optimal change rate of the total interaction energy in the LVRT process, the adjustment strategy of LVRT control parameters is constructed to meet voltage and damping requirements. Finally, a PMSG-connected system model is built on the MATLAB/Simulink platform to verify the effectiveness of the adjustment strategy. The results show that the proposed method can effectively improve the damping level under the fault transient condition, as well as supporting system voltage.

## Introduction

In recent years, LVRT control has been incorporated into the basic functions of wind turbines to ensure stable operation during failures and provide certain reactive power support for the power grid. In this case, the LVRT control will be coupled with other control links which may change the damping characteristics of PMSG-connected system during fault periods and cause oscillation stability problem. On August 9, 2019, a large-scale power failure occurred in the United Kingdom^1–4^. The LVRT control was applied immediately, while broadband oscillation was excited after that. The oscillations continually spread out during the LVRT process causing the instability of the system. The problem sounded the alarm for the oscillation stability of wind turbines during LVRT periods. Therefore, it is urgent to explore the coupling mechanism of LVRT control and oscillation stability of the system, and propose a parameter adjustment strategy that could both provide voltage support and meet system damping requirements.

Currently, oscillation stability analysis of the PMSG-connected system mainly adopts the impedance analysis method, the eigenvalue analysis method or the energy function method. The impedance analysis method is based on the impedance models of the power electronic devices and the other controllers and components in the system. It uses the Nyquist stability criterion to analyze the system stability and predict the potential resonant frequency and stability margin of the system^5–9^. Reference^9^ used the impedance method to construct a small-signal model of wind turbine, and analyzed that increasing active current injection is detrimental to system stability. The eigenvalue analysis method identifies the stability of system by analyzing the real part of the eigenvalue of the system state equation, and reveals the dominant mode and key influencing factor that causes system instability according to the participation factor method^10,11^. This method can obtain abundant information such as the oscillation mode, the damping characteristic, the participation factor, and the sensitivity, etc., so it is widely applied in the analysis of oscillatory stability. Reference^12^ analyzed the stability of wind power connected system using modal analysis, and the results showed that the instability during LVRT is mainly affected by the phase locked loop. Reference^13^ used modal analysis for comparative analysis and concluded that the stability analysis that ignores the dynamic changes in the rotor current reference value of the wind turbine may be inaccurate. However, since modern power systems usually have a high variable dimension, the application of the eigenvalue analysis method is limited by modeling. The energy function method calculates the dynamic energy of the interconnected system by building the transient energy function, and then assesses the stability of the system according to Lyapunov’s second stability law. This method can analyze the stability margin of the system and the influence of parameter variation, and it has been applied to analyzing the transient stability of traditional power system. References^14–16^ was based on the transient energy function, and it located the oscillation source by analyzing the energy variation characteristics of different branches in the network. Compared with other methods, the energy function method has a broad definition of energy attributes, in which the interaction energy can portray the energy coupling trajectory between different control links.

In view of the oscillation suppression of the PMSG connected system during LVRT, it can be normally divided into three categories according to the suppression principles, namely optimizing controller parameters, changing control topology, and adding damping. The main idea of the first method^17–20^ is to change the control parameters of each subsystem of wind turbines by means of collaborative optimization, to maximize the damping ratio, and finally to change the oscillation resonance position of the system. The above method can fundamentally eliminate the instability risk of the system. In the second method, by changing the control topology structure^21–25^, additional branches are added on the basis of the existing control system, so as to change the operation mode of PMSG and improve the damping level of the system. The advantage of this method is that it can realize online real-time oscillation suppression, and has a good suppression effect for different types of oscillation. The suppression effect of additional suppression branch will be greatly reduced, and the economy will be greatly reduced by adding the suppression device again. The third suppression method^26–28^ is to add an independent damping device in the PMSG, and add damping control branches considering observability and controllability requirements of the system, so as to realize the oscillation online damping control. However, all of the above-mentioned research ignored the influence of LVRT process on system stability, and taking damping as a single control objective, which is difficult to realize both voltage stability and oscillation suppression.

In order to analysis the cause of the oscillation of PMSG connected system during LVRT process, an interaction energy analysis method is proposed in this paper and the parameter adjustment method is constructed based on that. Firstly, component connection method (CCM) is used to construct a modular state-space model of PMSG-WPIS under fault transient condition, and the dynamic energy function of the system is constructed with the first integration method, based on which, the dynamic energy flow path of PMSG is described under the LVRT control. Then, the energy interaction process between LVRT control and other subsystems is analyzed, and the contribution of LVRT control to the interaction energy is quantified, and the coupling mechanism of voltage support and damping characteristics in the process is expounded. On this basis, aiming at the optimal change rate of the total interactive energy in the fault transient process, the LVRT control parameter adjustment strategy is constructed in coordination with voltage support and damping requirements. Finally, a PMSG-connected system model is built on the MATLAB/Simulink platform to verify the effectiveness of the strategy.

## Dynamic energy model of PMSG-WPIS during LVRT

### Control architecture of PMSG-WPIS

**Figure 1:**
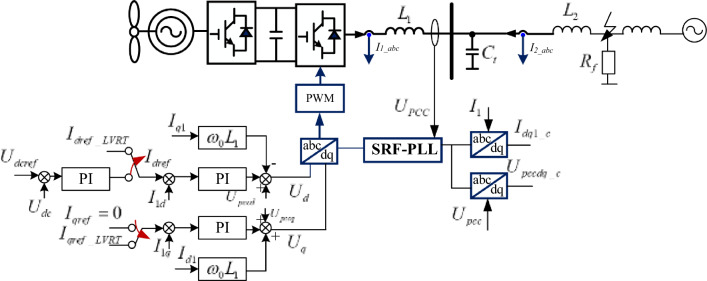
Structure of PMSG-WPIS.

During LVRT periods, the equivalent circuit and control architecture of PMSG-WPIS are shown in Fig. 1. When a large disturbance fault occurs in the AC power grid side, the converter of PMSG in rotor side will disconnect the outer control loop of DC voltage and adopt LVRT control in the fault transient process.

The current inner loop control of PMSG is characterized as follows^29^:

1 $$ \left\{ \begin{gathered} u_{sd}^{{}} = K_{pd} \left( {i_{dref} - i_{d1} } \right) + K_{id} \int {\left( {i_{dref} - i_{d1} } \right)dt} - \omega_{0} Li_{q1} + Ri_{d1} + u_{pccd} \hfill \\ u_{sq}^{{}} = K_{pd} \left( {i_{qref} - i_{q1} } \right) + K_{id} \int {\left( {i_{qref} - i_{q1} } \right)dt} + \omega_{0} Li_{d1} + Ri_{q1} + u_{pccq} \hfill \\ \end{gathered} \right. $$

where *K*_*idq*_ and *K*_*pdq*_ are the integral coefficients and the proportional coefficients of dq-axis current loop control respectively. *R* and *L* are the filter resistance and filter inductance of the converter in grid side respectively. *i*_*d*1_ and *i*_*q*1_ are the current output of the converter in grid side in d and q axis respectively. *i*_*dref*_ and *i*_*qref*_ are the current reference values of the d and q axis respectively, the output of which are controlled by LVRT during the fault stage. *u*_*sd*_ and *u*_*sq*_ are the voltage of the inverter terminal point in d and q axis respectively. *u*_*pccd*_ and *u*_*pccq*_ are the voltages of the point of common coupling (PCC) in d and q axis respectively. *ω*_*0*_ is the synchronous angular frequency, the value of which is equal to 100*π*.

The system will disconnect the voltage outer loop after the voltage of PCC falls. Then, the LVRT strategy is adopted, and the current control command, i.e.* i*_*dref*_ and *i*_*qref*_ are obtained according to the voltage drop degree. The reference value of the current in LVRT control is depicted as follows:

2 $$ \left\{ \begin{gathered} i_{qref} = - K\left( {0.9 - U_{pcc} } \right)I_{N} \hfill \\ i_{dref} = \sqrt {I_{\max }^{2} - i_{qref}^{2} } \hfill \\ \end{gathered} \right. $$

where, *K* represents reactive current compensation coefficient, the value of which is 1.5 in this paper. *U*_*pcc*_ represents per unit voltage of PCC. *I*_*N*_ is the rated current. *I*_*max*_ is the maximum allowable current value of the grid-side converter in the fault stage.

In PMSG, phase locked loop (PLL) controller is used to track the voltage phase of PCC. The transfer function of a PLL can be expressed as:

3 $$ u_{pccq} \left( {K_{pp} + \frac{{K_{ip} }}{s}} \right) = s\theta_{pll} $$

where, *u*_*pccq*_ is the q-axis voltage of PCC, *K*_*p_pll*_ and *K*_*i_pll*_ are the PI controller ratio and integration parameter of PLL. *θ*_*pll*_ is the output voltage phase of PLL.

### Modular state-space model of PMSG-WPIS

In order to quantitatively analyze the influence of the interaction of each control loop on the stability of power system during LVRT, an energy interaction analysis method is proposed. This method can divide the PMSG connected system into multiple subsystems, and use the dynamic energy function to establish the quantitative correlation between several subsystems, so as to realize the stability evaluation of the whole system.

According to Eqs. (1)–(3) and the control structure of the PMSG control system, the modular state space model of PMSG connected system can be divided by CCM^30^, into five sub-systems. They are d-axis current inner loop sub-system and q-axis current inner loop sub-system, PLL sub-system, grid side d-axis sub-system and q-axis sub-system. Among the above sub-systems, there is a mutual coupling state quantity between each sub-system.

The state space model of each subsystem can be shown as follows.

- **D-axis current innerloop sub-system**
  The state space model of LVRT d-axis subsystem can be described by Eq. (4).
  4 $$ \left\{ {\begin{array}{*{20}l} {\frac{1}{{K_{id} }}\frac{{d\Delta U_{cd} }}{dt}{ = } - \cos (\theta_{0} )\Delta I_{d1} + F_{d1} } \hfill \\ \begin{gathered} L_{1} \frac{{d\Delta I_{d1} }}{dt}{ = } - K_{pd} \Delta I_{d1} + \cos (\theta_{0} )\Delta U_{cd} + F_{d2} \hfill \\ F_{d1} = \sqrt {I_{\max }^{2} - \Delta I_{q}^{*} } - \sin (\theta_{0} )\Delta I_{q1} - I_{q10} \Delta \theta_{pll} \hfill \\ F_{d2} = \left( \begin{gathered} K_{p} \cos (\theta_{0} )\sqrt {I_{\max }^{2} - \Delta I_{q}^{*} } - K_{p} \sin (\theta_{0} )\Delta I_{q}^{*} \hfill \\ - \sin (\theta_{0} )\Delta U_{cq} - (K_{p} I_{q10} + U_{q0} )\Delta \theta_{pll} + \omega_{0} L_{1} \Delta I_{q1} - \Delta U_{pccd} \hfill \\ \end{gathered} \right) \hfill \\ \end{gathered} \hfill \\ \end{array} } \right. $$
  where *K*_id_ and *K*_pd_ are the current inner loop integration gain coefficient and proportion gain coefficient in d-axis. *L*_1_ and *R*_1_ represent the equivalent reactance and resistance from the terminal point to the PCC. *θ*_0_ is the steady-state value of the phase difference between PCC voltage (i.e. ***U***_pcc,dq_) and fault point voltage (i.e. ***U***_m,dq_). *I*_*q*10_, *U*_*q*0_ and *I*_*q*1_c0_ represent the steady-state values of ***I***_dq,1_, ***U***_dq_ and ***I***_dq,1c_ in q-axis, respectively.* U*_*cd*_ and *U*_*cq*_ are the intermediate state variables of current inner loop control in dq-axis, respectively. *I*_*d*1_ and *I*_*q*1_ are the current components of PMSG terminal point in d-axis and q-axis, respectively. *I*_*dref*_ and *I*_*qref*_ are the reference value of current inner loop control in d-axis and q-axis, respectively. *U*_*pccd*_ and *U*_*pccq*_ represent the voltage components of PCC in d-axis and q-axis, respectively. Δ characterizes the amount of change during fault periods. The subscript 0 indicates the steady-state component.
- **Q-axis current innerloop sub-system**
  The state space model of LVRT q-axis subsystem can be described as:
  5 $$ \left\{ {\begin{array}{*{20}l} \begin{gathered} \frac{1}{{K_{iq} }}\frac{{d\Delta U_{cq} }}{dt}{ = } - \cos (\theta_{0} )\Delta I_{q1} + F_{q2} \hfill \\ L_{1} \frac{{d\Delta I_{q1} }}{dt}{ = } - (K_{pq} + R_{1} )\Delta I_{q1} + \cos (\theta_{0} )\Delta U_{cq} + F_{q2} \hfill \\ \end{gathered} \hfill \\ \begin{gathered} F_{q1} = \Delta I_{qref} + \sin (\theta_{0} )\Delta I_{d1} + I_{d10} \Delta \theta_{pll} \hfill \\ F_{q2} = \left( \begin{gathered} K_{pq} \sin (\theta_{0} )\sqrt {I_{\max }^{2} - \Delta I_{q}^{*} } + K_{pq} \cos (\theta_{0} )\Delta I_{q}^{*} \hfill \\ + \sin (\theta_{0} )\Delta U_{cd} + (K_{pq} I_{d10} + U_{d0} )\Delta \theta_{pll} \hfill \\ - \omega_{0} L_{1} \Delta I_{d1} - \Delta U_{pccq} \hfill \\ \end{gathered} \right) \hfill \\ \end{gathered} \hfill \\ \end{array} } \right. $$
  where *K*_iq_ and *K*_pq_ are the current inner loop integration gain coefficient and proportion gain coefficient in q-axis. *I*_*d*10_, *U*_*d*0_ and *I*_*d*1_c0_ represent the steady-state values of ***I***_dq,1_, ***U***_dq_ and ***I***_dq,1c_ in d-axis.
- **PLL subsystem**
  The state space model of PLL subsystem can be described as:
  6 $$ \left\{ {\begin{array}{*{20}l} {\frac{1}{{K_{i\_pll} }}\frac{{d\Delta x_{pll} }}{dt} = - \Delta \theta_{pll} + F_{pll1} } \hfill \\ {\frac{{d\Delta \theta_{pll} }}{dt} = - K_{p\_pll} \Delta \theta_{pll} + \Delta x_{pll} + F_{pll2} } \hfill \\ {F_{pll1} = - \frac{{U_{pccq0} }}{{U_{pccd0}^{2} + U_{pccq0}^{2} }}\Delta U_{pccd} + \frac{{U_{pccd0} }}{{U_{pccd0}^{2} + U_{pccq0}^{2} }}\Delta U_{pccq} } \hfill \\ {F_{pll2} = K_{p\_pll} ( - \frac{{U_{pccq0} }}{{U_{pccd0}^{2} + U_{pccq0}^{2} }}\Delta U_{pccd} + \frac{{U_{pccd0} }}{{U_{pccd0}^{2} + U_{pccq0}^{2} }}\Delta U_{pccq} )} \hfill \\ \end{array} } \right. $$
  where, *K*_*i_pll*_ and *K*_*p_pll*_ are the PI control integration gain coefficient and proportion gain coefficient of PLL, respectively. *x*_*p_pll*_ is the intermediate state variable of PLL. *U*_*pccd*0_ and *U*_*pccq*0_ represent the steady-state values of*** U***_pcc,dq_ in d-axis and q-axis.
- **Equivalent grid side D-axis subsystem**
  The state space model of equivalent grid side d-axis subsystem can be described as:
  7 $$ \left\{ {\begin{array}{*{20}l} {{\text{C}}_{t} \frac{{d\Delta U_{sd} }}{dt} = - \Delta I_{d2} + F_{sd1} } \hfill \\ {L_{2} \frac{{d\Delta I_{d2} }}{dt} = - R_{2} \Delta I_{d2} + \Delta U_{ctd} + F_{sd2} - R_{f} \Delta I_{d2} } \hfill \\ {F_{sd1} { = }\Delta I_{d1} + \omega_{0} C_{t} \Delta U_{sq} } \hfill \\ \begin{gathered} F_{sd2} = \omega_{0} L_{2} \Delta I_{q2} \hfill \\ \Delta U_{pccd} = \Delta U_{sd} \hfill \\ \end{gathered} \hfill \\ \end{array} } \right. $$
  where, *C*_*t*_ indicates grounding capacitance of PCC, *L*_*2*_ is the equivalent reactance between PCC and fault point, *I*_*d*2_,* I*_*q*2_ are the current components between PCC and fault point in d-axis and q-axis, respectively. Δ*I*_*d*2_ indicates the perturbation value of ***I***_dq,2_ in d-axis.
- **Equivalent grid side Q-axis subsystem**
  The state space model of equivalent grid side q-axis subsystem can be described as:
  8 $$ \left\{ {\begin{array}{*{20}l} {{\text{C}}_{t} \frac{{d\Delta U_{{{\text{sq}}}} }}{dt} = - \Delta I_{q2} + F_{sq1} } \hfill \\ {L_{2} \frac{{d\Delta I_{q2} }}{dt} = - R_{2} \Delta I_{q2} + \Delta U_{ctq} + F_{sq2} - \Delta I_{q2} R_{f} } \hfill \\ {F_{sq1} = \Delta I_{q1} - \omega_{0} C_{t} \Delta U_{sd} } \hfill \\ \begin{gathered} F_{sq2} = - \omega_{0} L_{2} \Delta I_{d2} \hfill \\ \Delta U_{pccq} = \Delta U_{sq} \hfill \\ \end{gathered} \hfill \\ \end{array} } \right. $$
  where $R_{f}$ is the transition resistance.
  Therefore, the above five sub-system expressions can be depicted as:
  9 $$ \left\{ {\begin{array}{*{20}l} {C\frac{d\Delta U}{{dt}}{ = } - K_{L} \Delta I{\text{ + F}}_{C} } \hfill \\ {L\frac{d\Delta I}{{dt}}{ = } - K_{R} \Delta I + K_{C} \Delta U + {\text{F}}_{L} } \hfill \\ \end{array} } \right. $$
  where, the left differential coefficients of Eqs. (4)–(8) are uniformly expressed as *C*, *L* and *K*_R_. *K*_C_ and *K*_L_ are constant terms. State variables are uniformly represented as Δ*U* and Δ*I*, in which, the interaction links that affect the voltage (i.e. Δ*U*) are uniformly represented as *F*_C_ and the interaction links that affect the voltage (i.e. Δ*I*) are uniformly represented as* F*_L_.

### Dynamic energy function of PMSG connected system

Based on the energy function construction method proposed in Ref.^31^, the corresponding dynamic energy model can be obtained via cross-multiplying the two expressions in Eq. (9) and integrating the time *t*, shown as follows:

10 $$ \begin{gathered} \frac{1}{2}CK_{C} \Delta U^{2} { + }\frac{1}{2}LK_{L} \Delta I^{2} + K_{L} K_{R} \int {\Delta I^{2} } dt \hfill \\ - K_{C} \int {F_{C} } \Delta Udt - K_{L} \int {F_{L} \Delta Idt} {\text{ = Constant}} \hfill \\ \end{gathered} $$

Based on Eq. (10), the energy function can be defined as:

11 $$ V = V_{s} - V_{d} - V_{{\text{t}}} $$

12 $$ \left\{ {\begin{array}{*{20}l} {V_{s} { = }\frac{1}{2}C \cdot K_{C} \cdot \Delta U^{2} { + }\frac{1}{2}L \cdot K_{L} \cdot \Delta I^{2} } \hfill \\ {V_{d} = - K_{L} K_{R} \int {\Delta I^{2} } dt} \hfill \\ {V_{t} = K_{C} \int {F_{C} } \Delta Udt + K_{L} \int {F_{L} \Delta Idt} } \hfill \\ \end{array} } \right. $$

where, *V*_*s*_ is the stored energy, *V*_*d*_ is dissipated energy on the resistance *K*_*R*_, and *V*_t_ represents the interaction energy between subsystems.

By calculating the partial derivative of the energy function *V* with respect to time *t* in Eq. (12), the result is shown in Eq. (13). It can be seen that the derivative of the energy function *V* with respect to time *t* is zero, proving that the system is energy conserved.

13 $$ \begin{aligned} \dot{V} & = \frac{\partial V}{{\partial \Delta U}} \cdot \frac{d\Delta U}{{dt}} + \frac{\partial V}{{\partial \Delta I}} \cdot \frac{d\Delta I}{{dt}} + \frac{\partial V}{{\partial t}} \\ & = \left( {K_{C} F_{C} \cdot \Delta U - K_{L} K_{C} \Delta U \cdot \Delta I} \right) + \left( {K_{L} F_{L} \cdot \Delta I + K_{L} K_{C} \cdot \Delta U\Delta I - K_{L} K_{R} \Delta I^{2} } \right) \\ & \quad + \left( {K_{L} K_{R} \cdot \Delta I^{2} - K_{C} F_{C} \cdot \Delta U - K_{L} F_{L} \cdot \Delta I} \right) = 0 \\ \end{aligned} $$

Based on the Lyapunov's second principle^14,32^, when the system is disturbed, the state variable would deviate from the equilibrium point, and the system would obtain a certain stored energy. If the stored energy gradually decays over time, the amplitude of the oscillation will become smaller and smaller, and when the equilibrium state is reached, the amplitude of the oscillation is zero, then at this equilibrium point, the system is asymptotically stable. On the contrary, if the storage energy of the system is increasing, then at this equilibrium point, the system is unstable. If the stored energy of the system is neither increased nor consumed, then the system is in a critically stable state at this equilibrium point.

As can be seen from Eq. (13), in an energy conservation system, the change rate of stored energy is composed of dissipative energy and interactive energy change rate. In other words, the stability of the system is mainly composed of the dissipative action of the system itself and the dissipative action generated by the interaction between control links. Among them, the dissipated energy of the system is always negative, that means a positive damping effect, and the change rate of the stored energy is mainly determined by the change rate of the interaction energy. Therefore, the contribution of each interaction link to the system stability can be evaluated by exploring the interaction energy change rate among subsystems.

## Interaction among subsystems of PMSG connected system during LVRT

### Dynamic energy flow path of PMSG connected system during LVRT

Take Eqs. (4)–(8) into Eq. (9), the expression of dynamic energy among subsystems can be derived. The interaction energy component of each subsystem is shown as follows.

**Figure 2:**
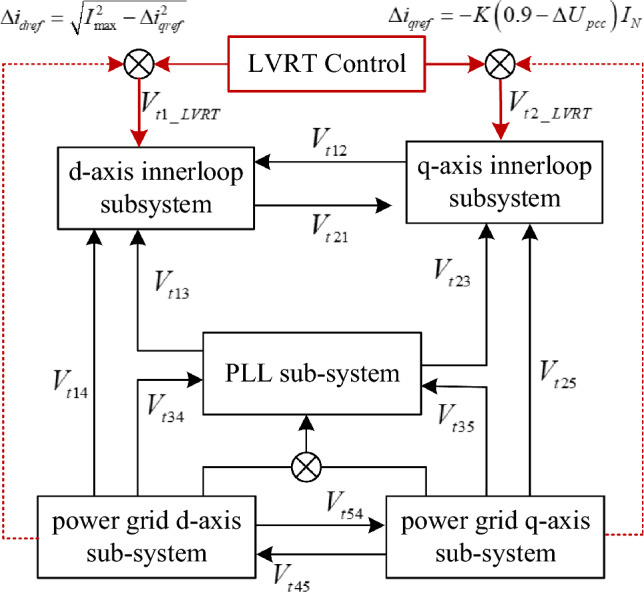
The interaction energy flow in the PMSG connected system during LVRT.
  In Fig. 2, the arrow direction represents the energy transferred from the starting subsystem to the endpoint subsystem. As the red line shows that, LVRT control will introduce a new interaction energy channel between current loop subsystems and the grid side subsystems, which may increase the risk of PMSG-grid coupling induced oscillation. Moreover, since the asymmetric features of LVRT control, the dq coupling effect is strengthened which may change the dynamic characteristics of the PMSG during LVRT. As a result, it is necessary to explore the coupling mechanism between system low voltage support and damping stability, in order to provide a theoretical basis for subsequent parameter adjustment.

- **D-axis current inner loop subsystem**
  The interaction energy component of d-axis current inner loop subsystem can be described as follows:
  14 $$ \begin{aligned} & V_{t1} = V_{t1\_LVRT} + V_{t12} + V_{t13} + V_{t14} \\ & \left\{ {\begin{array}{*{20}l} {V_{t1\_LVRT} = \int {\cos \theta_{0} \sqrt {I_{\max }^{2} - \Delta I_{qref}^{2} } \Delta U_{cd} dt} + \int {K_{pd} \cos^{2} \theta_{0} \sqrt {I_{\max }^{2} - \Delta I_{qref}^{2} } \Delta I_{d1} dt} } \hfill \\ {V_{t12} = - \int {\sin \theta_{0} \cos \theta_{0} \Delta I_{q1} \Delta U_{cd} dt} - \int {K_{pd} \sin \theta_{0} \cos \theta_{0} \Delta I_{qref} \Delta I_{d1} dt} } \hfill \\ {\quad - \int {\sin \theta_{0} \cos \theta_{0} \Delta U_{cq} \Delta I_{d1} dt} + \int {\omega_{0} L_{1} \cos \theta_{0} \Delta I_{q1} \Delta I_{d1} dt} } \hfill \\ {V_{t13} = - \int {\cos \theta_{0} \left( {K_{pd} I_{q10} + U_{q0} } \right)\Delta \theta_{pll} \Delta I_{d1} dt} - \int {I_{q10} \cos \theta_{0} \Delta \theta_{pll} \Delta U_{cd} dt} } \hfill \\ {V_{t14} = - \int {\cos \theta_{0} \Delta U_{pccd} \Delta I_{d1} dt} } \hfill \\ \end{array} } \right. \\ \end{aligned} $$
  where* V*_*t1_LVRT*_ is the interaction energy between LVRT and d-axis current inner loop subsystem. *V*_*t*12_ is the interaction energy between q-axis current inner loop subsystem and d-axis current inner loop subsystem. *V*_*t*13_ is the interaction energy between PLL and d-axis current inner loop subsystem. *V*_*t*14_ is the interaction energy between d-axis grid side subsystem and d-axis current inner loop subsystem.
  It can be seen from Eq. (14) that, after LVRT is introduced, a new dq-axis coupling energy channel is generated, which is mainly influenced by the current inner loop control parameter and LVRT control parameters.
- **Q-axis current inner loop subsystem**
  The interaction energy component of q-axis current inner loop subsystem can be described as follows:
  15 $$ \begin{gathered} V_{t2} = V_{t22} + V_{t21} + V_{t23} + V_{t25} \hfill \\ \left\{ {\begin{array}{*{20}l} {V_{t2\_LVRT} = \int {\cos \theta_{0} \Delta I_{qref}^{{}} \Delta U_{cq} dt} + \int {R_{k} \cos^{2} \theta_{0} \Delta I_{qref}^{{}} \Delta I_{q1} dt} } \hfill \\ {\quad + \int {R_{k} \sin \theta_{0} \cos \theta_{0} \sqrt {I_{\max }^{2} - \Delta I_{qref}^{2} } \Delta I_{q1} dt} } \hfill \\ {V_{t21} = \int {\sin \theta_{0} \cos \theta_{0} \Delta I_{d1} \Delta U_{cq} dt} + \int {\sin \theta_{0} \cos \theta_{0} \Delta U_{cd} \Delta I_{q1} dt} } \hfill \\ {\quad - \int {\omega_{0} L_{1} \cos \theta_{0} \Delta I_{q1} \Delta I_{d1} dt} } \hfill \\ {V_{t23} = \int {\cos \theta_{0} \left( {K_{pq} I_{d10} + U_{d0} } \right)\Delta \theta_{pll} \Delta I_{q1} dt} + \int {I_{d10} \cos \theta_{0} \Delta \theta_{pll} \Delta U_{cq} dt} } \hfill \\ {V_{t25} = - \int {\cos \theta_{0} \Delta U_{pccq} \Delta I_{q1} dt} } \hfill \\ \end{array} } \right. \hfill \\ \end{gathered} $$
  where *V*_*t2_LVRT*_ is the interaction energy between LVRT and q-axis current inner loop subsystem. *V*_*t*21_ is the interaction energy between d-axis current inner loop subsystem and q-axis current inner loop subsystem. *V*_*t*23_ is the interaction energy between PLL and q-axis current inner loop subsystem. *V*_*t*14_ is the interaction energy between grid side q-axis subsystem and q-axis current inner loop subsystem.
  Comparing Eq. (14) with Eq. (15), after LVRT control is introduced, the interaction energy component between d-axis and q-axis presents asymmetric features.
- **PLL subsystem**
  The interaction energy component of PLL subsystem can be described as follows:
  16 $$ \begin{gathered} V_{t3} = V_{t34} + V_{t35} + V_{t345} \hfill \\ \left\{ {\begin{array}{*{20}l} {V_{t34} = \int { - K_{p\_pll} \frac{{U_{pccq0} }}{{U_{pccd0}^{2} + U_{pccq0}^{2} }}\Delta U_{pccd} \Delta \theta_{pll} dt} } \hfill \\ {V_{t35} = \int {K_{p\_pll} \frac{{U_{pccd0} }}{{U_{pccd0}^{2} + U_{pccq0}^{2} }}\Delta U_{pccq} \Delta \theta_{pll} dt} } \hfill \\ {V_{t345} = \int {K_{pll8} \Delta U_{pccq} \Delta U_{pccd} dt} } \hfill \\ \end{array} } \right. \hfill \\ \end{gathered} $$
  where *V*_*t*34_ is the interaction energy between grid side d-axis subsystem and PLL subsystem. *V*_*t*35_ is the interaction energy between grid side q-axis subsystem and PLL subsystem. *V*_*t*345_ is the interaction energy between the PLL and the coupling effect of d-axis and q-axis grid side subsystem.
- **The equivalent grid side d-axis subsystem**
  The interaction energy component of the equivalent grid side d-axis subsystem can be described as follows:
  17 $$ \begin{gathered} V_{t4} = V_{t41} + V_{t45} \hfill \\ \left\{ {\begin{array}{*{20}l} {V_{t41} = \int {\Delta I_{d1} \Delta U_{sd} dt} } \hfill \\ {V_{t45} = \int {\omega_{0} C_{t} \Delta U_{sd} \Delta U_{sq} dt} + \int {\omega_{0} L_{2} \Delta I_{q2} \Delta I_{d2} dt} } \hfill \\ \end{array} } \right. \hfill \\ \end{gathered} $$
  where* V*_*t4*1_ is the interaction energy between d-axis current inner loop subsystem and grid side q-axis subsystem. *V*_*t*45_ is the interaction energy between grid side d-axis subsystem and q-axis subsystem.
- **The equivalent grid side q-axis subsystem**
  The interaction energy component of the equivalent grid side q-axis subsystem can be described as follows:
  18 $$ \begin{gathered} V_{t5} = V_{t52} + V_{t54} \hfill \\ \left\{ {\begin{array}{*{20}l} {V_{t52} = \int {\Delta I_{q1} \Delta U_{ctq} dt} } \hfill \\ {V_{t45} = - \int {\omega_{0} C_{t} \Delta U_{ctd} \Delta U_{ctq} dt} - \int {\omega_{0} L_{2} \Delta I_{q2} \Delta I_{d2} dt} } \hfill \\ \end{array} } \right. \hfill \\ \end{gathered} $$
  where *V*_*t*52_ is the interaction energy between q-axis current inner loop subsystem and grid side d-axis subsystem. *V*_*t*54_ is the interaction energy between grid side q-axis subsystem and d-axis subsystem.
  According to the interaction energy component in Eqs. (14)–(18), the energy flow path among the control links in PMSG during LVRT can be depicted, as shown in Fig. 2.

### Analysis of the interaction between LVRT control and subsystems

This chapter focuses on exploring the interaction energy between LVRT control and the subsystems, and analyzing the impact of voltage support parameters on system stability.

It is depicted in Fig. 2 that, the Interaction energy component dominated by LVRT control is $V_{t1\_LVRT}$ and $V_{t2\_LVRT}$. Substituting Eq. (1) into Eqs. (14) and (15), the derivative of the two iteration energy terms can be deducted:

19 $$ \left\{ {\begin{array}{*{20}l} {\dot{V}_{t1\_LVRT} = \cos \theta_{0} \Delta I_{dref} \int {K_{pd} \left( {\Delta I_{dref} - \Delta I_{d1} } \right)dt} + K_{pd} \cos^{2} \theta_{0} \Delta I_{dref} \Delta I_{d1} } \hfill \\ {\dot{V}_{t2\_LVRT} = \cos \theta_{0} \Delta I_{qref} \int {K_{pq} \left( {\Delta I_{qref} - \Delta I_{q1} } \right)dt} + K_{pq} \cos^{2} \theta_{0} \Delta I_{qref} \Delta I_{q1} } \hfill \\ {\quad + K_{pq} \sin \theta_{0} \cos \theta_{0} \Delta I_{dref} \Delta I_{q1} } \hfill \\ \end{array} } \right. $$

Suppose that there induced sub/super-synchronous oscillation with a mode of α + jωc after fault. The expression of the current oscillation components in Eq. (19) can be expressed as:

20 $$ \left\{ {\begin{array}{*{20}l} {\Delta I_{qref} = e^{\alpha t} A_{ref} \cos \left( {\omega_{c} t + \theta_{Iref} } \right)} \hfill \\ {\Delta I_{dref} = e^{\alpha t} \sqrt {I_{\max }^{2} - A_{Iref}^{2} } \sin \left( {\omega_{c} t + \theta_{Iref} } \right)} \hfill \\ {\Delta I_{q1} = e^{\alpha t} A_{Iq1} \cos \left( {\omega_{c} t + \theta_{Iq} } \right)} \hfill \\ {\Delta I_{d1} = e^{\alpha t} A_{Id1} \cos \left( {\omega_{c} t + \theta_{Id} } \right)} \hfill \\ \end{array} } \right. $$

where *A*_*I*_ is the oscillation amplitude of the state variables. *θ*_*I*_ is the oscillation angle of the state variables.

Taking Eq. (20) into Eq. (19) and extracting the non periodic components can obtain that:

21 $$ \left\{ \begin{gathered} \dot{V}_{t1\_LVRT\_dc} = - \cos \theta_{0} K_{pd} \frac{1}{{\omega_{c}^{2} }}e^{2\alpha t} \sqrt {I_{\max }^{2} - A_{ref}^{2} } A_{Id1} \frac{{\cos \left( {\theta_{ref} - \theta_{d} } \right)}}{2} \hfill \\ \dot{V}_{t2\_LVRT\_dc} = K_{pq} \cos^{2} \theta_{0} e^{2\alpha t} A_{ref} A_{Iq1} \frac{{\cos \left( {\theta_{ref} - \theta_{q} } \right)}}{2} \hfill \\ \end{gathered} \right. $$

where $\dot{V}_{t1\_LVRT\_dc}$ and $\dot{V}_{t2\_LVRT\_dc}$ are the non periodic components of the interaction energy between LVRT control and dq-axis current innerloop subsystem respectively.

It can be seen from Eq. (21), that the trend of $\dot{V}_{t1\_LVRT\_dc}$ and mainly determined by the amplitude and phase of the dq-axis current and its reference. Combined with Eq. (2), the oscillation amplitude of the dq-axis reference is related to the voltage of PCC, expressed as:

22 $$ \left\{ {\begin{array}{*{20}c} {A_{\Delta ref} = KI_{N} A_{\Delta upcc} } \\ {\theta_{\Delta ref} = \theta_{\Delta upcc} } \\ \end{array} } \right. $$

where $A_{\Delta upcc}$ and $\theta_{\Delta upcc}$ are the oscillation amplitude and phase of PCC voltage, which is mainly determined by the voltage drop depth.

From Eq. (22), with the same voltage drop scenario, the larger $K$ is, the greater the oscillation amplitude $A_{\Delta ref}$.

Furthermore, the relationship between the oscillation amplitude of $I_{d1}$ and $I_{q1}$ is analyzed. According to the variable transmission paths in control links in Fig. 1, the transfer function from dq-axis reference to $I_{d1}$ and $I_{q1}$ can be written as:

23 $$ \Delta I_{dq1} = G_{12} \left( s \right)\Delta I_{dqref} = \frac{1}{{1 + G_{I} G_{L} }}\Delta I_{dqref} $$

where $G_{I} = k_{pwm} \left( {K_{pdq} + \frac{{K_{idq} }}{s}} \right)$ is the dq-axis transfer function of the current inner loop. Since the control structure of the dq-axis current inner loop is similar, the control parameters are expressed as $K_{pdq}$ and $K_{idq}$ uniformly. $G_{L} = \frac{1}{{L_{1} s + R_{1} }}$ is the transfer function of the dq-axis current and voltage on the transmission line.

As seen from Eq. (23), the relationship between $\Delta I_{dq1}$ and the oscillation amplitude of the dq-axis current reference can be expressed as:

24 $$ \left\{ {\begin{array}{*{20}c} {A_{\Delta Idq1} = A_{\Delta dqref} \times \left| {G_{12} \left( s \right)} \right|} \\ {\theta_{\Delta Idq1} - \theta_{\Delta dqref} = \angle G_{12} \left( s \right)} \\ \end{array} } \right. $$

Combining Eqs. (23) and (24), $\left| {G_{12} \left( s \right)} \right|$ is determined by the dq-axis current innerloop parameters $K_{pdq}$. With the increase of $K_{pdq}$, the value of it decreases. The phase of $\angle G_{12} \left( s \right)$ lies in $\left( { - \frac{\pi }{2},\frac{\pi }{2}} \right)$.

Take Eqs. (22) and (24) into Eq. (21), the analytical expressions for the current inner loop parameters, LVRT control parameters, and interaction energy change rate can be obtained:

25 $$ \left\{ \begin{gathered} \dot{V}_{t1\_LVRT\_dc} = - \cos \theta_{0} K_{pd} \frac{1}{{\omega_{c}^{2} }}e^{2\alpha t} \left( {I_{\max }^{2} - A_{\Delta upcc}^{2} I_{N}^{2} K^{2} } \right)\left| {G_{12d} \left( s \right)} \right|\frac{{\cos \angle G_{12d} \left( s \right)}}{2} \hfill \\ \dot{V}_{t2\_LVRT\_dc} = K_{pq} \cos^{2} \theta_{0} e^{2\alpha t} A_{\Delta upcc}^{2} I_{N}^{2} K^{2} \left| {G_{12q} \left( s \right)} \right|\frac{{\cos \angle G_{12q} \left( s \right)}}{2} \hfill \\ \end{gathered} \right. $$

where $G_{12d} \left( s \right)$ and $G_{12q} \left( s \right)$ are the transfer function of $G_{12} \left( s \right)$ with d-axis and q-axis parameters.

It can be seen from Eq. (25), that the change rate of the interaction energy between LVRT control and the d-axis current inner loop subsystem $V_{t1\_LVRT}$ is constantly negative. It means that the interaction process has a positive dissipation effect on the system oscillation, which does well to the downward of the energy accumulation process during LVRT. The change rate is mainly determined by the proportional gains of the d-axis current inner loop control *K*_*pd*_ and the reactive power compensation coefficient *K* of LVRT control. Decreasing *K*_*pd*_ will accelerate the dissipation of the interaction energy $V_{t1\_LVRT}$ and improve the system stability during LVRT. However, increasing the *K* parameter may reduce the dissipation effect generated by the d-axis current inner loop and the LVRT control link, which is not conducive to system stability.

The change rate of the interaction energy between LVRT control and the q-axis current inner loop subsystem $V_{t2\_LVRT}$ is constantly positive, promoting the accumulation of energy during LVRT and intensifying system oscillation divergence. It is also a key interaction link in triggering system oscillation and divergence. According to Eq. (25), decreasing the proportional gains of q-axis current inner loop control *K*_*pq*_ and increasing the reactive power compensation coefficient *K* of LVRT control will accelerate the upwards of $V_{t2\_LVRT}$, harming the system stability.

Comparing $V_{t1\_LVRT}$ and $V_{t2\_LVRT}$, since the value of $\omega_{c}^{ - 2}$ is relatively small, the change rate of $V_{t2\_LVRT}$ plays the dominant role in the interaction between LVRT and the other subsystems. If the optimal voltage support is taken as the single objective and the control parameters such as reactive power compensation coefficient is set at the maximum boundary, the system may go unstable.

As a result, the setting of the LVRT control parameters and current inner loop control parameters should consider both system voltage support and damping requirements simultaneously, in order to achieve effective voltage support while ensuring damping requirements.

## Parameter adjustment method coordinated with voltage support and damping requirements

### Objective function

It can be concluded from the above analysis that during LVRT periods, the increase of current inner loop control parameters and reactive power compensation coefficient will intensify the interaction between LVRT control and q-axis current inner loop subsystem, accelerating the energy accumulation in LVRT. If the parameter setting is unreasonable, the system may be induced to oscillate and destabilize. Therefore, from the perspective of interaction energy path optimization, this section aims to reduce the negative dissipation energy generated by the interaction between LVRT control and the q-axis current inner loop subsystem, and improve dissipation rate of the overall interaction energy. Thus, a parameter adjustment strategy during LVRT process is established.

According to the energy conservation function constructed by Eqs. (11)–(13), it can be concluded that the trend of stored energy in the system is mainly determined by interaction energy $V_{t}$. When the $\dot{V}_{t}$ is negative, the stored energy gradually decreases. The smaller the $\dot{V}_{t}$ is, the higher the stability level of the system will be. Therefore, the minimum changing rate of interaction energy $\dot{V}_{t}$ is taken as the parameter optimization objective.The objective function is constructed as:

26 $$ \min \dot{V}_{t\_sum} = f\left( {K_{pd} ,K_{pq} ,K} \right) $$

27 $$ \dot{V}_{t\_sum} = \sum\limits_{i = 1}^{5} {\dot{V}_{ti} } $$

where $f\left( \cdot \right)$ is the correlation expression between the control parameters and the interaction energy change rate, which can be obtained from Eqs. (14)–(18).

### Constraints

- **Constraints for supporting transient voltage**
  In the LVRT process, reactive voltage support and effective LVRT capability are still the main control objectives of PMSG. Therefore, the requirements of transient voltage support should be mainly met^33^, that is shown as follows:
  28 $$ I_{qref}^{{}} \ge 1.5\left( {0.9 - u_{g} } \right)I_{N} $$
- **Current inner loop control parameter constraints**
  The current inner loop control is a typical second-order system, considering establishing constraints on the damping ratio of the second-order closed-loop transfer function composed of the current inner loop:
  29 $$ 0.5 < \xi_{i} < 0.8 $$
  where $\xi_{i}$ is the damping ratio of current innerloop control, which can be expressed as:
  30 $$ \xi_{i} = \frac{{K_{p} }}{{2\sqrt {K_{i} L} }} $$
  Then, the constraint of *K*_*i*_ can be deduced:
  31 $$ \frac{{0.390625K_{p}^{2} }}{L} < K_{i} < \frac{{K_{p}^{2} }}{L} $$
- **Reactive current compensation coefficient constraints**
  The value of the reactive current compensation factor *K* in LVRT control is related to the limiting link of the inverter, which is generally taken from 1.2 to 1.5. Thus, the constraint of *K* is shown as below:
  32 $$ 1.2 < K < 1.5 $$

### Optimization solution method

According to the above objective function and constraint conditions, the parameter optimization model of PMSG-WPIS in LVRT stage can be constructed. It can be seen that the parameter optimization model in this article only contains three variables, and there is a linear correlation between the objective function and the variables. Based on the aforementioned stability analysis results, the search direction of the variables has been preliminarily determined. The problem to be solved has the characteristics of fewer independent variables, a linear objective function, and a relatively fixed search direction. Therefore, a basic algorithm, the pattern search method^34^, is applied to determine the control parameters.

The solution method contains several following steps.

***Step 1.*** The port voltage of PMSG is measured online. When voltage drop occurs, the value of voltage drop is measured at first. Then, the current inner loop proportional control coefficient *K*_*p*_, current outer loop integral control coefficient *K*_*i*_, PLL proportional control coefficient *K*_*pp*_ and reactive current compensation coefficient *K* of PMSG-WPIS are collected when the fault occurs initially. The initial value of optimization coefficient is determined and recorded as the initial solution *k*_1_, which has the description as follows:

33 $$ k_{1} = \left[ {K_{pd}^{\left( 1 \right)} ,K_{pq}^{\left( 1 \right)} ,K^{\left( 1 \right)} } \right] $$

***Step 2.*** It is determined that whether the initial value meets the constraint conditions. If the conditions are met, *k*_1_ is the current optimal solution; if not, *k*_1_ is re-determined until a feasible solution is found.

***Step 3.*** On the basis of the current optimal solution (e.g. $k_{i}$, *i* = 1, 2, …), a new feasible solution is obtained by applying pattern search to update the control parameter, which can be recorded as $k_{i + 1}$. By repeatedly calculate the objective function, the optimal solution can be found according to the comparing rule. If objective function is satisfied by $V_{i} > V_{i + 1}$, $k_{i + 1}$ is the current feasible solution. Otherwise, repeat step 3.

***Step 4.*** Repeat the search process until the number of iterations is satisfied and the search is terminated. The current optimal solution is the optimal LVRT control parameter.

It should be noted that the selection of the pattern search method is mainly because the optimization model proposed in this paper requires fewer control parameters to be optimized, and the search direction and scope are relatively fixed. The direct search method is suitable for the solution. However, when optimizing the parameters of multiple wind farms, algorithms such as differential evolution (DE)^35^ and particle swarm optimization (PSO)^36^ can be used to improve the search accuracy of key parameters, obtaining more precise optimal solutions. Since the optimization algorithm is not the focus, it will not be elaborated here.

## Simulation verification

To verify the correctness of the adjustment strategy mentioned in this paper, the PMSG connected system simulation model, shown as Fig. 1, is built in MATLAB/Simulink. Analyze the oscillation stability of the systems during fault with the scenario set in Ref.^31^. The rated capacity of the PMSG is 1 MW, which is connected to the PCC through a 0.69/20 kV on-site transformer and then a 20/230 kV transformer. The parameters setting of PMSG grid side control is shown in Table 1. Considering the symmetry and stability of the PMSG control system, the dq axis current inner loop control parameters in the simulation model adopt the same settings.

**Table 1:** The parameters of PMSG connected system.

Parameters	Symbol	Value
Current inner loop proportional gain	$k_{ip\_g}$	1.05
Current inner loop integral gain	$k_{ii\_g}$	40.2
Phase locked loop proportional gain	$k_{pll.p}$	10
Phase locked loop integral gain	$k_{pll.i}$	40
Transmission line inductance	$L_{F}$	0.45
Transmission line resistance	$R_{F}$	0.12
The reactive power compensation coefficient	$K$	1.5

### Verification of interaction energy characteristics in different cases

To verify the accuracy of the interaction energy analysis method proposed in this paper, oscillation divergence and convergence simulation cases are set during fault LVRT, and the changes in the total interaction energy of the system are calculated separately.

**Figure 3:**
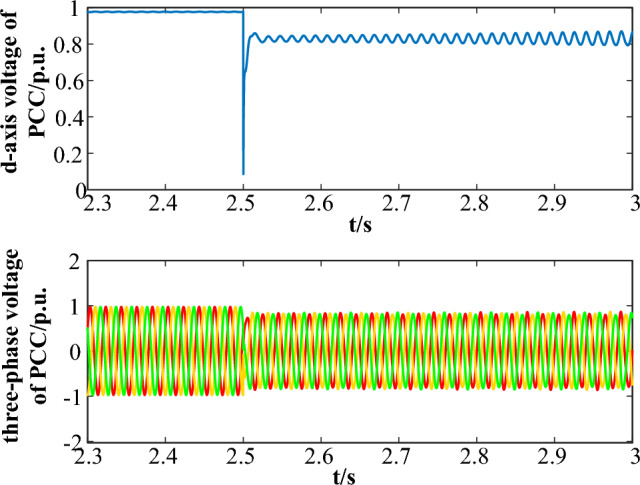
The oscillation curve in Case 1.
  Collect the oscillation component of the PMSG after the fault occurs, and calculate the trend of the total interaction energy of the system and the interaction energy dominated by LVRT control with the oscillation trajectory. The result is shown in Fig. 4.

- **Oscillation divergence case**
  The oscillation divergence curve is shown in Fig. 3. At 2.5 s, a three-phase short circuit fault occurred at the end of PMSG, causing the voltage to drop to 0.8 p.u.. Then, the LVRT control was put into operation, causing system oscillation and it gradually goes divergent.

**Figure 4:**
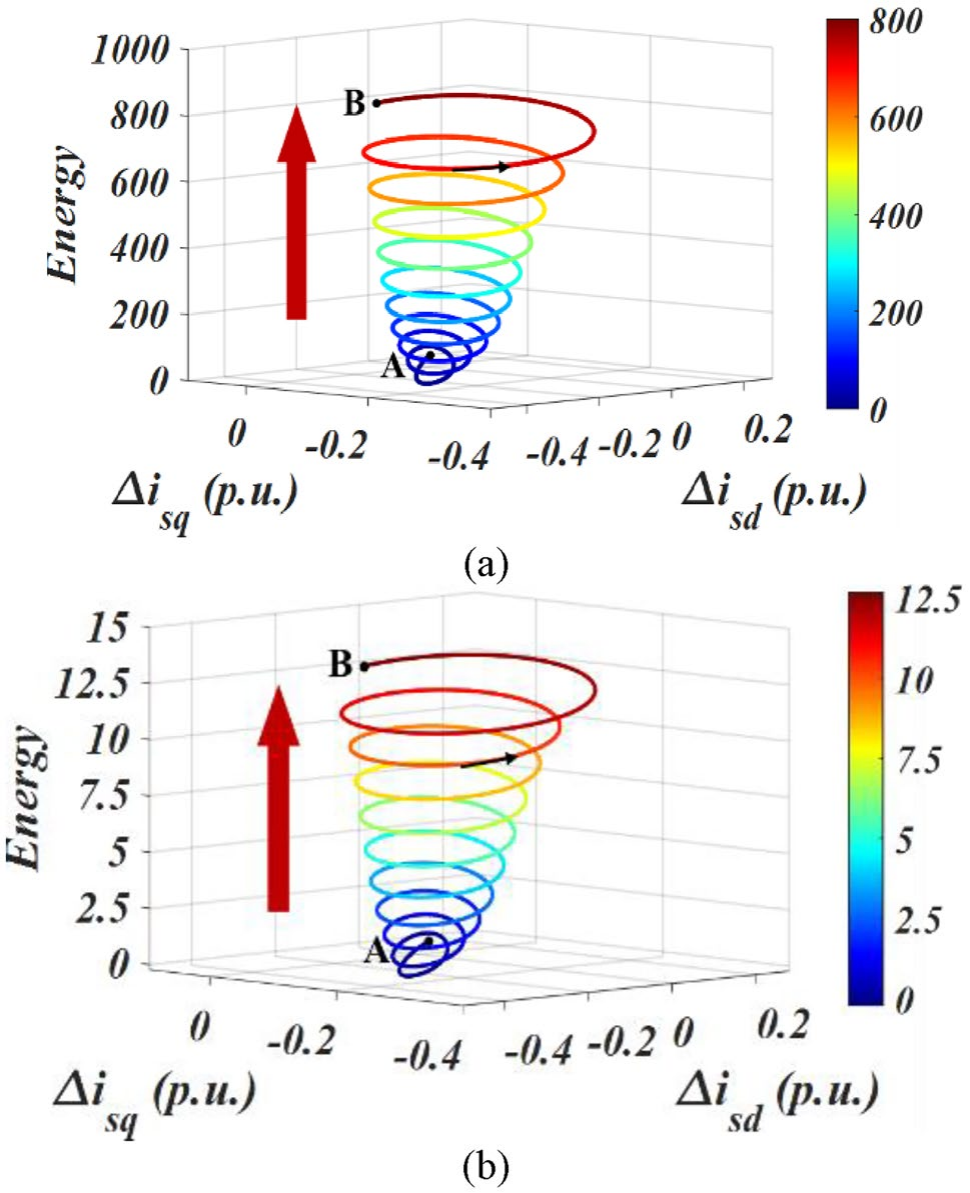
The interaction energy oscillation trajectory in Case 1. (**a**) The total interaction energy of the system. (**b**) The interaction energy dominated by LVRT control.
  Figure 4a depicts the variation of the total interaction energy of the system with the oscillation trajectory (from A to B). Accompanied by oscillation divergence, the total interaction energy of the system gradually increases, showing a trend of outward divergence and spiral growth, indicating that the accumulated energy of the system continues to increase after being disturbed, and the system gradually goes unstable. The result is consistent with time-domain simulation.
  The interaction energy between LVRT control and q-axis current inner loop control subsystems is depicted in Fig. 3b. It is depicted that the interaction energy is constantly positive, accelerating the accumulation of the system energy, which does harm to the system stability.
- **Oscillation convergence case**
  The oscillation divergence curve is shown in Fig. 5. At 2.5 s, a three-phase short circuit fault occurred at the end of PMSG, causing the voltage to drop to 0.85 p.u.. Then, the LVRT control was put into operation, causing system oscillation and it gradually goes convergent.

**Figure 5:**
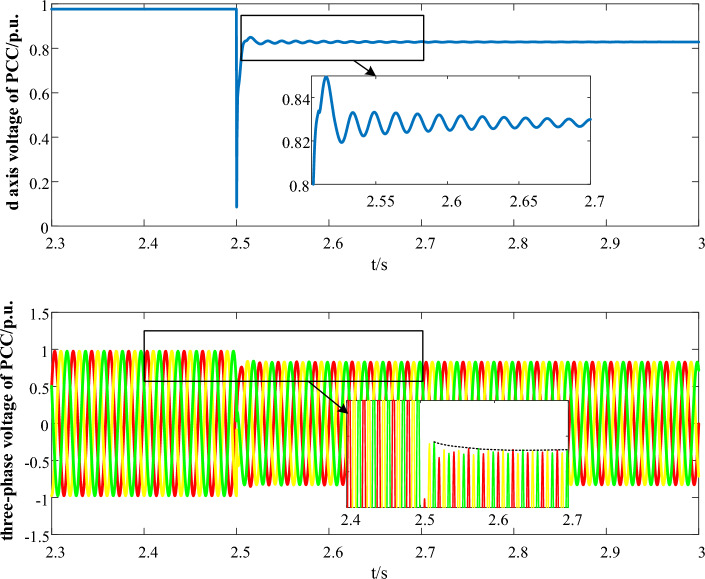
The oscillation curve in Case 2.
  Collect the oscillation component of the PMSG after the fault occurs, and according to Eqs. (14)–(18), calculate the trend of the total interaction energy in Case 2 with the oscillation trajectory, as shown in Fig. 6.

**Figure 6:**
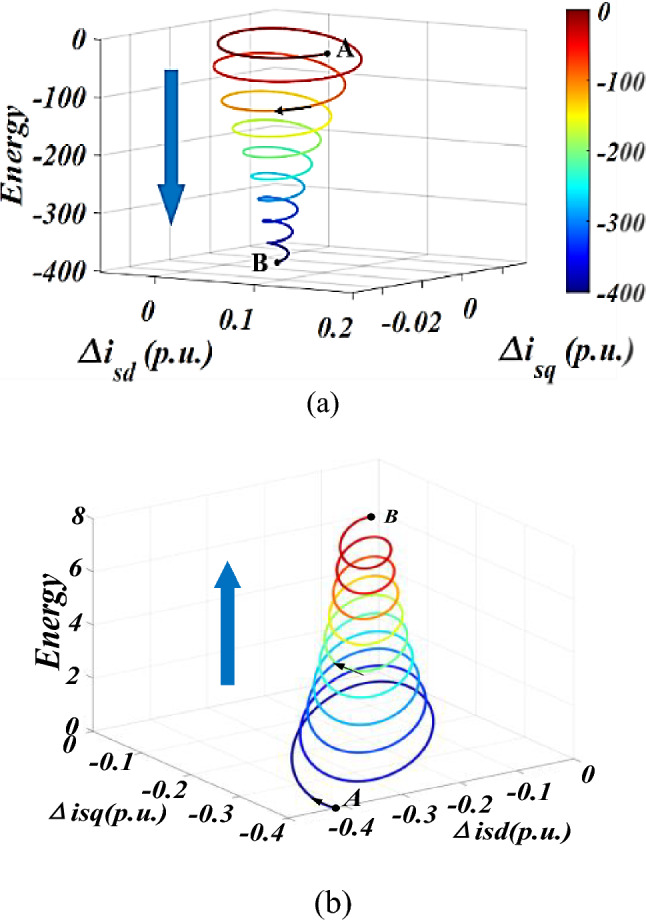
The interaction energy oscillation trajectory in Case 2. (**a**). The total interaction energy of the system; (**b**) The interaction energy dominated by LVRT control.
  The total interaction energy with the oscillation trajectory is shown in Fig. 6a. As the oscillation converges, the total interaction energy of the system shows a spiral downward trend, and the dynamic energy generated by the disturbance is gradually dissipated, leading to a stable system.
  Furthermore, the interaction energy between LVRT control and q-axis current innerloop subsystems is calculated as shown in Fig. 6b. The interaction energy generated by LVRT control remains positive and spirals upwards, exhibiting a negative dissipative effect on the system. However, due to the fact that the dissipative effect generated by the interaction between other subsystems in the system is greater than that generated by the LVRT control, the system exhibits an oscillatory convergence trajectory. Therefore, the overall interaction energy exhibits a spiral contraction feature.

### Verification of the influence of the parameters on the oscillation stability during LVRT

**Figure 7:**
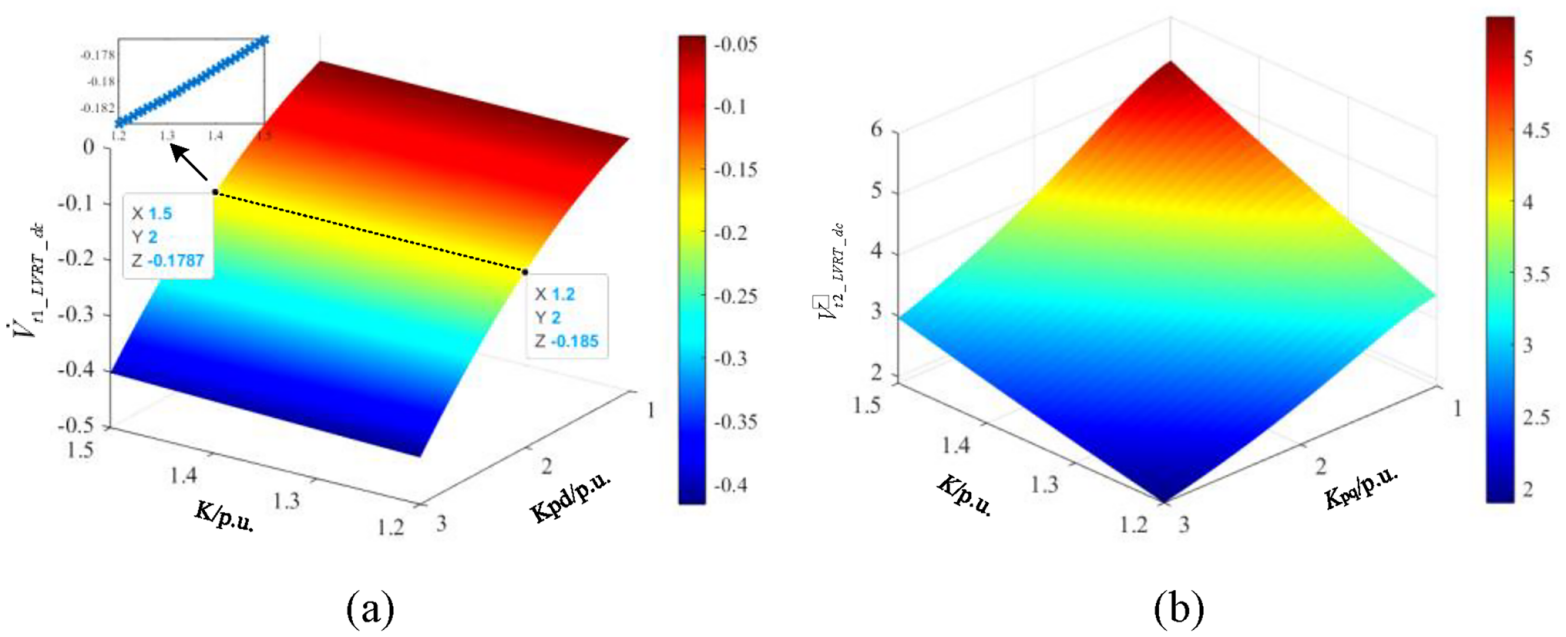
The change rate of interaction energy with different current inner loop parameters and LVRT control parameters.

Taking the oscillation divergence scenario as an example, substituting the parameters shown in Table 1 into Eq. (25), the influence of different current inner loop parameters and LVRT control parameters on $\dot{V}_{t1\_LVRT\_dc}$ and $\dot{V}_{t2\_LVRT\_dc}$ are calculated as shown in Fig. 7.

The change rate of interaction energy with different $K_{pd}$ and *K* is depicted in Fig. 7a. $\dot{V}_{t1\_LVRT\_dc}$ is negative, indicating that the interaction energy branch has a dissipative effect on system oscillation, which helps to stabilize the system. Meanwhile, increasing the proportional gain coefficient of the d-axis current inner loop $K_{pd}$ is beneficial for increasing the dissipation effect of the interaction energy branch and promoting system stability. The influence of LVRT control parameter *K* is relatively small. With the partial zoom of the figure, increasing LVRT parameter *K* will alleviate the dissipation effect of the energy branch, harming the system's stability.

The influence of the current inner loop parameter $K_{pd}$ and LVRT control parameter *K* is shown in Fig. 7b. $\dot{V}_{t2\_LVRT\_dc}$ is positive, whose value is larger than that of $\dot{V}_{t1\_LVRT\_dc}$ in Fig. 7a. Therefore, the interaction between the q-axis current inner loop and the LVRT control loop plays a dominant role and exhibits a negative dissipation, exacerbating the system oscillation divergence. Meanwhile, decreasing $K_{pd}$ or increasing LVRT parameter *K* will accelerate the accumulation of energy.

**Figure 8:**
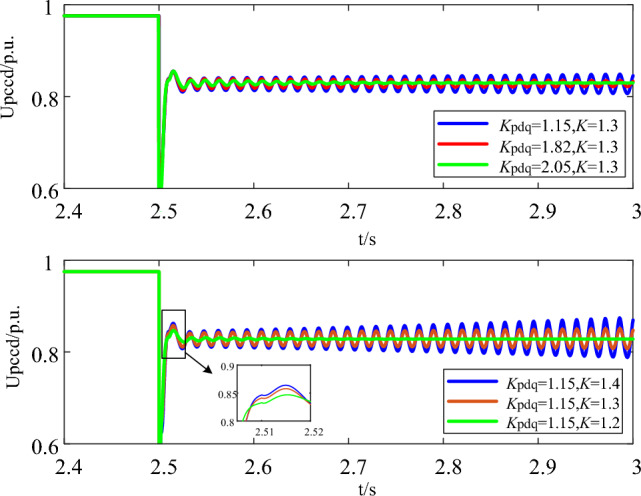
Simulation results with different current inner loop control parameters and LVRT control parameters.

Furthermore, time-domain simulation verification with different current inner loop control parameters and LVRT control parameters is carried out. The simulation result is shown in Fig. 8.

It can be seen in Fig. 8a, that increasing the control parameters of the current inner loop will intensify the negative dissipation effect, leading to increased oscillation divergence. From the zoomed part in Fig. 8b, increasing the LVRT control parameters can quickly provide transient reactive voltage support after fault, while the system stability will be sacrificed. With the LVRT control parameter going upward, the tends to converge from divergence. The simulation results are consistent with the theoretical derivation conclusion.

### Verification of parameter optimization control strategy

**Figure 9:**
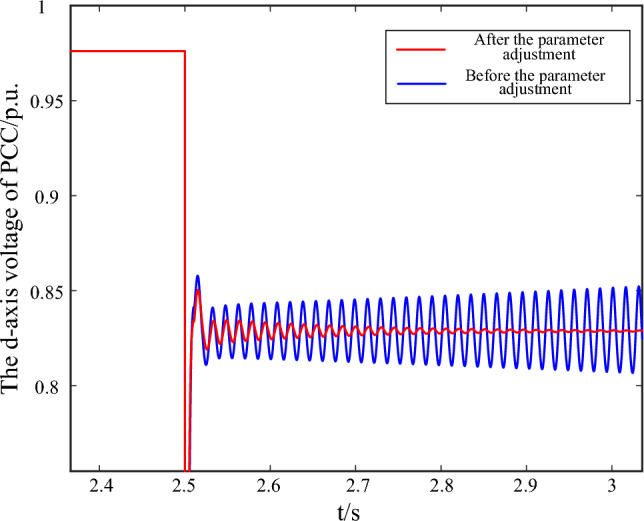
The oscillation curve of d-axis PCC voltage before and after parameter adjustment.

Taking the oscillation divergence scenario as an example, when t = 2.5 s, a voltage drop disturbance occurs at the transmission line, and the PCC voltage drops to 80% of the normal voltage value. The control parameters before optimization are: $K_{pd} = 1.05$,$K = 1.5$. After optimization, the control parameters are adjusted as: $K_{pd} = 2.11$,$K = 1.2$. The voltage waveforms of d-axis voltage of PCC before and after control parameter optimization are shown in Fig. 9.

The oscillation curve before optimization is shown as the blue line. The three-phase fault occurs at 2.5 s, and then the PCC voltage experienced a significant drop, causing the d-axis 63 Hz oscillation. If reasonable parameter adjustments are not made, the system will exhibit a divergent oscillation trend and gradually become unstable. After parameter optimization, the oscillation curve is shown by the red line, and the system oscillation quickly converges and gradually stabilizes. Since the reactive power compensation coefficient is adjusted lower, at the initial stage of fault, the degree of voltage recovery is smaller than the original parameter scheme. However, it still meet the requirement of voltage support while suppressing the system oscillation.

**Figure 10:**
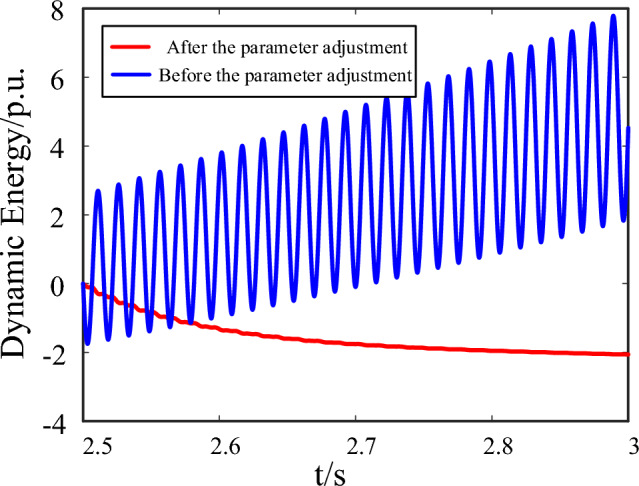
The dynamic energy of the system before and after parameter adjustment.

Furthermore, the system dynamic energy before and after parameter adjustment is shown as Fig. 10. From the starting time of fault occurrence, the total dynamic energy generated by the interaction among subsystems before parameter adjustment shows an increasing trend and gradually diverges. After parameter optimization, the interaction among subsystems shows a decreasing trend, which helps to quickly dissipate the accumulation energy during LVRT and achieve stable state of the system.

**Figure 11:**
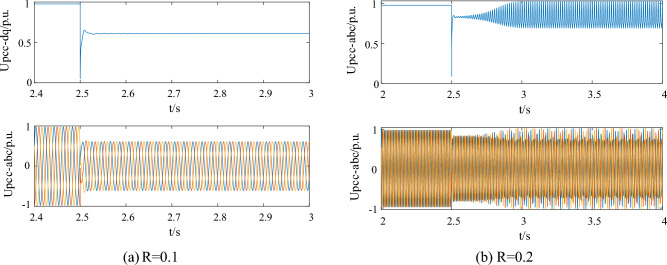
Simulation results with the parameter setting in Ref.^37^. (**a**) R = 0.1 (**b**) R = 0.2.

**Figure 12:**
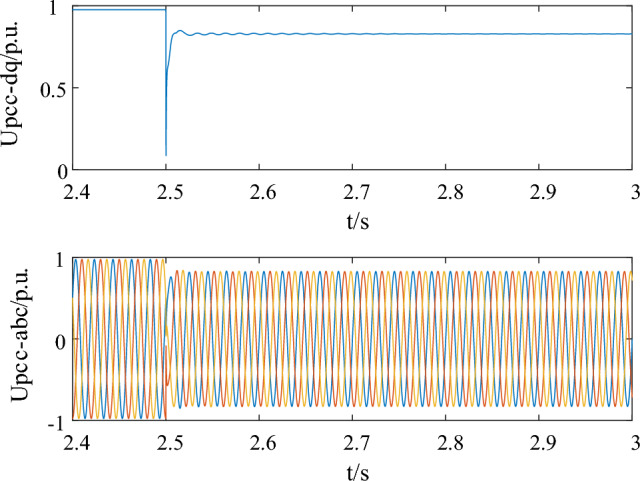
Simulation results with the proposed method in the case of R = 0.2.

Moreover, the method proposed in this article will be compared and validated with the existing LVRT strategy. Take the strategy in Ref.^37^ into the simulation model. When the grounding resistance was 0.1 p.u, the voltage curve is shown in Fig. 11a. The system did not excite oscillation and the strategy in Ref.^37^ gave sufficient support for the voltage. However, when the fault grounding resistance is 0.2, which is a common scenario during operation, the strategy proposed in the reference will stimulate system oscillation and gradually diverge, going unstable, as shown in Fig. 11b. This is because the control strategy in the reference ignores the impact of LVRT control on system oscillation stability. The simulation result in this case, with the parameters optimized by the proposed method in this paper, is depicted in Fig. 12. The oscillation could be suppressed quickly while ensuring voltage support, guaranteeing the stable operation of the wind power system.

## Conclusion

Aiming at the problem of oscillation instability in PMSG connected system during LVRT periods, this paper constructs an interaction energy analysis method and parameter adjustment strategy of PMSG. The main conclusions can be highlighted as follows.

1. The interaction energy model of the PMSG connected system constructed in this paper can quantify the contribution degree of the interaction between each control link to the system stability. If the change rate of interaction energy between the control links is positive, it indicates that the interaction has a negative damping contribution to the system. It will aggravate the accumulation of fault energy and be unfavorable to system stability. Conversely, if the change rate of interaction energy is negative, the interaction has a positive damping contribution to the system.
2. During the LVRT stage, the interaction between LVRT control and d-axis current inner loop control presents a positive dissipation effect, which is conducive to the rapid convergence of system oscillation. However, the interaction between LVRT control and q-axis current inner loop control presents a negative dissipation effect. With the increase of reactive power compensation coefficient and the decrease of current inner loop proportional gain coefficient, the accumulation energy during LVRT will be accelerated, which may lead to system oscillation divergence.
3. The parameter adjustment strategy proposed in this paper realizes the system oscillation suppression with consideration of voltage support requirements. By optimizing the interaction energy dissipation rate, the proposed strategy can effectively improve the oscillation stability of PMSG during LVRT process.

## Data Availability

All data generated or analyzed during this study are included in this published article.
